# Outcomes According to MSKCC Risk Score with Focus on the Intermediate-Risk Group in Metastatic Renal Cell Carcinoma Patients Treated with First-Line Sunitinib: A Retrospective Analysis of 2390 Patients

**DOI:** 10.3390/cancers12040808

**Published:** 2020-03-27

**Authors:** Ondrej Fiala, Jindrich Finek, Alexandr Poprach, Bohuslav Melichar, Jindrich Kopecký, Milada Zemanova, Katerina Kopeckova, Tomas Mlcoch, Tomas Dolezal, Lenka Capkova, Tomas Buchler

**Affiliations:** 1 Department of Oncology and Radiotherapeutics, Faculty of Medicine and University Hospital in Pilsen, Charles University, alej Svobody 80, 304 60 Pilsen, Czech Republic; finek@fnplzen.cz; 2 Biomedical Center, Faculty of Medicine in Pilsen, Charles University, alej Svobody 76, 304 60 Pilsen, Czech Republic; 3 Department of Comprehensive Cancer Care, Masaryk Memorial Cancer Institute, Zluty kopec 7, 656 53 Brno, Czech Republic; poprach@mou.cz; 4 Department of Comprehensive Cancer Care, Faculty of Medicine, Masaryk University, Kamenice 5, 625 00 Brno, Czech Republic; 5 Department of Oncology, Palacky University Medical School and Teaching Hospital, I.P. Pavlova 6, 775 20 Olomouc, Czech Republic; bohuslav.melichar@fnol.cz; 6 Department of Oncology, University Hospital in Hradec Králové, Sokolská 581, 50005 Hradec Králové, Czech Republic; kopecjin@fnhk.cz; 7 Department of Oncology, First Faculty of Medicine, Charles University and General University Hospital, U Nemocnice 499/2, 128 08 Prague, Czech Republic; Milada.Zemanova@vfn.cz; 8 Department of Oncology, Second Faculty of Medicine, Charles University and Motol University Hospital, V Uvalu 84, 150 06 Prague, Czech Republic; katerina.kopeckova@fnmotol.cz; 9 Institute of Health Economics and Technology Assessment (iHETA), Vaclavska 316/12, 120 00 Prague, Czech Republic; mlcoch@iheta.org (T.M.); dolezal@iheta.org (T.D.); 10 Faculty of Medicine, Masaryk University, Department of Pharmacology, Kamenice 753/5, 625 00 Brno, Czech Republic; 11 Institute of Biostatistics and Analyses, Ltd., Poštovská 68/3, 602 00 Brno, Czech Republic; capkova@biostatistika.cz; 12 Department of Oncology, First Faculty of Medicine, Charles University and Thomayer University Hospital, Videnska 800, 140 59 Prague, Czech Republic; tomas.buchler@ftn.cz

**Keywords:** renal cell carcinoma, Memorial Sloan–Kettering Cancer Center risk, sunitinib, first line, outcome

## Abstract

Background: The Memorial Sloan–Kettering Cancer Center (MSKCC) prognostic model has been widely used for the prediction of the outcome of metastatic renal cell carcinoma (mRCC) patients treated with systemic therapies, however, data from large studies are limited. This study aimed at the evaluation of the impact of the MSKCC score on the outcomes in mRCC patients treated with first-line sunitinib, with a focus on the intermediate-risk group. Methods: Clinical data from 2390 mRCC patients were analysed retrospectively. Progression-free survival (PFS), overall survival (OS), and objective response rate (ORR) were analysed according to the MSKCC risk score. Results: ORR, median PFS, and OS for patients with one risk factor were 26.7%, 10.1, and 28.2 months versus 18.7%, 6.2, and 16.2 months, respectively, for those with two risk factors (ORR: *p* = 0.001, PFS: *p* < 0.001, OS: *p* < 0.001). ORR, median PFS, and OS were 33.0%, 17.0, and 44.7 months versus 24.1%, 9.0, and 24.1 months versus 13.4%, 4.5, and 9.5 months in the favourable-, intermediate-, and poor-risk groups, respectively (ORR: *p* < 0.001, PFS: *p* < 0.001, OS: *p* < 0.001). Conclusions: The results of the present retrospective study demonstrate the suitability of the MSKCC model in mRCC patients treated with first-line sunitinib and suggest different outcomes between patients with one or two risk factors.

## 1. Background

Renal cell carcinoma (RCC) represents a common malignancy, with incidence increasing in developed countries [1,2]. The management of metastatic RCC (mRCC) has been markedly changing in recent years with the introduction of targeted therapies and immune checkpoint inhibitors, leading to significant improvements in patient survival. Several prognostic models have been proposed for the prediction of outcome of mRCC patients treated with systemic therapies. The Memorial Sloan–Kettering Cancer Center (MSKCC) prognostic model developed by Motzer et al. has been widely used in clinical trials as well as in the common clinical practice. In this model, patients are classified based on the number of risk factors into three prognostic groups, that is, favourable-, intermediate-, and poor-risk [3].

Antiangiogenic tyrosine kinase inhibitors, such as sunitinib and pazopanib, had been shown to be safe and effective in MSKCC favourable- or intermediate-risk patients, and were established as a standard of care in the first-line systemic therapy [4,5]. Despite its worldwide use, there has been limited number of large studies comparing efficacy with regard to the MSKCC score in mRCC patients treated with targeted agents outside of clinical trials. About 50–60% of all mRCC patients are classified as MSKCC intermediate-risk. This group comprises a heterogeneous population of patients with regard to the type and number of risk factors. 

In the present retrospective registry-based study, we analysed the impact of MSKCC classification on the outcomes in a large cohort of mRCC patients treated with first-line sunitinib in the real-life clinical practice, with a focus on the intermediate-risk group.

## 2. Materials and Methods

### 2.1. Study Design and Treatment 

The present study is a retrospective registry-based analysis of adult mRCC patients treated with first-line sunitinib between May 2006 and January 2018. Patients who had received chemotherapy or cytokines prior to sunitinib were not included. Progression-free survival (PFS), overall survival (OS), and objective response rate (ORR) were analysed in the entire cohort and based on the number of risk factors in the MSKCC intermediate-risk group. Sunitinib (Sutent; Pfizer Inc, New York, USA) was administered orally at the standard approved dosing until disease progression, unacceptable toxicity, or patient refusal. Temporary discontinuation or dose reductions for toxicity followed clinical practice guidelines. Subsequent anticancer therapy after progression was at the discretion of the treating physicians.

### 2.2. Data Source

The data were obtained from the renal cell carcinoma information system (RENIS) registry that includes data on approximately 95% of mRCC patients treated with targeted therapy in the Czech Republic [6]. The RENIS registry, initiated in 2007, provides retrospective anonymised data on patient baseline clinical characteristics as well as on previous therapies for mRCC, laboratory parameters, treatment course and outcomes, and toxicity that are updated twice a year (http://renis.registry.cz). The RENIS registry and the use of registry data for analysis were approved on 15 May 2013 by the Multicentre Ethics Committee of the Masaryk Memorial Cancer Institute in Brno, Czech Republic. The patients signed informed consent with the inclusion of their data in the registry.

### 2.3. Outcome Assessment 

The clinical status of the patients was assessed continuously during the course of treatment. Physical examination and routine laboratory tests were performed at least every six weeks, and computed tomography (CT) was performed every three to four months during the treatment. The objective tumour response was assessed locally by the attending physician using Response Evaluation Criteria in Solid Tumors (RECIST) version 1.1 in terms of: complete response (CR), partial response (PR), stable disease (SD), and progressive disease (PD); ORR included patients achieving CR and PR [7].

### 2.4. Statistical Analysis

Data were described by absolute and relative frequencies for categorical variables and median for quantitative variables. Statistical differences in ORR were calculated using Pearson’s chi-square test; for >2 groups (i.e., good-, intermediate-, and poor-risk), Bonferroni correction for multinomial testing was performed so as to adjust *p*-values. OS was defined as the time from sunitinib treatment initiation to the date of death due to any cause. PFS was defined as the time from sunitinib treatment initiation to the date of first documented progression or death of any cause. Survival analysis for OS and PFS was conducted using the Kaplan–Meier method complemented by the 95% confidence interval (95% CI) for estimates of probability survival. Statistical significance of differences in survival among subgroups was assessed using the log-rank test. Univariable Cox proportional hazards models were used to evaluate the effect of individual risk factors on the survival measures. Hazard ratios (HRs) were calculated with 95% confidence interval and the statistical significance of hazard ratios was assessed by means of the Wald test. All statistical tests were performed at a significance level of α = 0.05 (two groups) and α = 0.025 (three groups Bonferroni correction) (all tests two-sided). Analysis was performed in the SPSS software (IBM Corp. Released 2016. IBM SPSS Statistics for Windows, Version 24.0.0.1 Armonk, NY: IBM Corp.) and software R version 3.6.0 (www.r-project.org).

## 3. Results

### 3.1. Baseline Characteristics 

**Figure 1:**
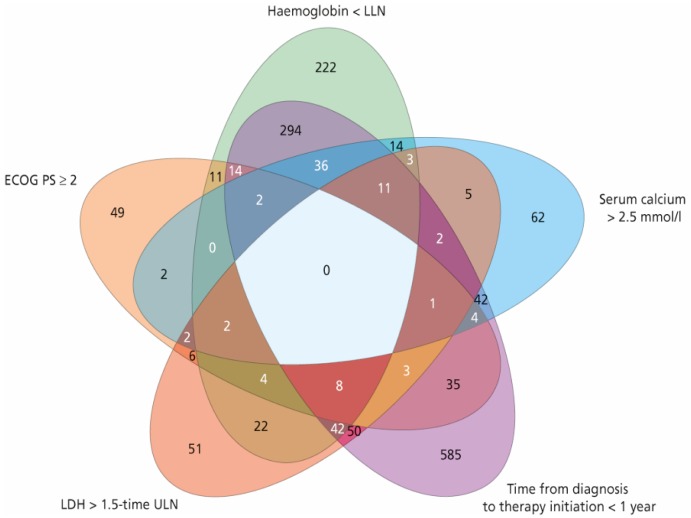
Frequency diagram of each combination of risk factors for the Memorial Sloan–Kettering Cancer Center (MSKCC) score. ECOG PS = Eastern Cooperative Oncology Group performance status, LLN = lower limit of normal, ULN = upper limit of normal, LDH = lactate dehydrogenase.

A total of 2390 patients with mRCC were treated with first-line sunitinib, including 806, 1450, and 134 patients in the favourable, intermediate, and poor MSKCC risk group, respectively. In the intermediate-risk group, 969 patients had one risk factor and 481 patients had two risk factors. Patient characteristics, including a detailed distribution of MSKCC score within the respective MSKCC risk group, are summarised in Table 1. A frequency diagram of each combination of risk factors for the MSKCC score is shown in Figure 1.

### 3.2. Treatment Outcomes 

**Figure 2:**
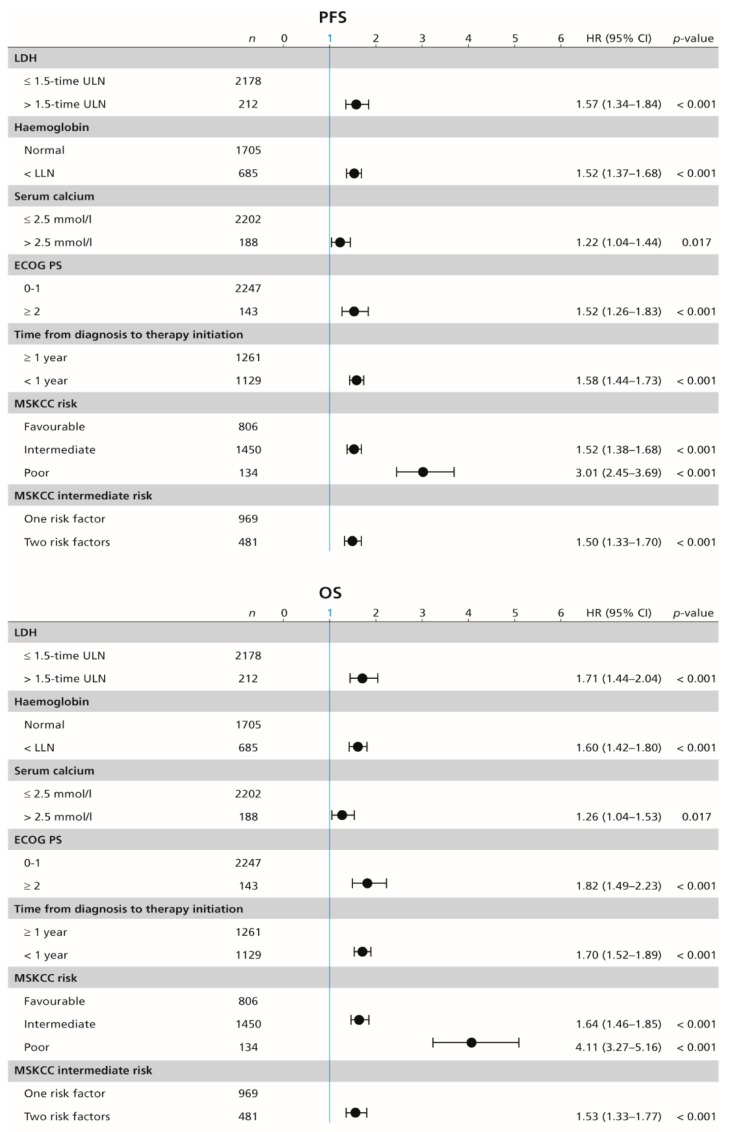
Forest plot (univariable Cox proportional-hazard regression model) showing the association between survival and MSKCC risk factors. MSKCC = Memorial Sloan–Kettering Cancer Center (MSKCC) score, PFS = progression-free survival, OS = overall survival, *n* = number of included patients, ECOG PS = Eastern Cooperative Oncology Group performance status, ULN = upper limit of normal, LDH = lactate dehydrogenase, HR = hazard ratio, CI = confidence interval.

In the whole cohort, median PFS and OS were 10.6 (95% CI 9.9–11.5) months and 28.5 (95% CI 26.3–30.5) months, respectively, with ORR of 26.5%. Each of the individual MSKCC risk factors show significant association with PFS and OS in the univariable Cox proportional-hazards regression; that is, high serum lactate dehydrogenase (LDH) (HR 1.57; 95% CI 1.34–1.84; *p* < 0.001, and 1.71; 95% CI 1.44–2.04; *p* < 0.001, respectively), haemoglobin concentration below the lower limit of normal (HR 1.52; 95% CI 1.37–1.68; *p* < 0.001, and 1.60; 95% CI 1.42–1.80; *p* < 0.001, respectively), serum calcium concentration above the upper limit of normal (HR 1.22; 95% CI 1.04–1.44; *p* = 0.017, and 1.26; 95% CI 1.04–1.53; *p* = 0.017, respectively), ECOG PS ≥ 2 (HR 1.52; 95% CI 1.26–1.83; *p* < 0.001, and 1.82; 95% CI 1.49–2.23; *p* < 0.001, respectively), and time from diagnosis to the initiation of systemic therapy of less than one year (HR 1.58; 95% CI 1.44–1.73; *p* < 0.001, and 1.70; 95% CI 1.52–1.89; *p* < 0.001, respectively; Figure 2).

**Figure 3:**
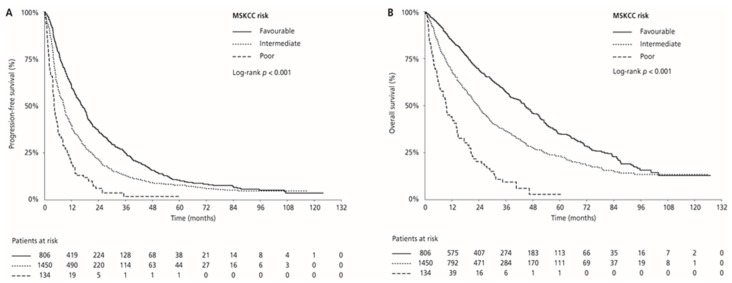
Kaplan–Meier estimates of progression-free survival (PFS) (**A**) and overall survival (OS) (**B**) according to the MSKCC risk category.

**Figure 4:**
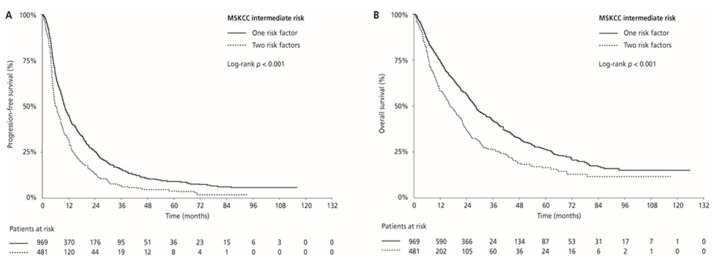
Kaplan–Meier estimates of progression-free survival (PFS) (**A**) and overall survival (OS) (**B**) according to the number of risk factors in the MSKCC intermediate-risk group.

Median PFS and OS were 17.0 (95% CI 15.4–18.8) and 44.7 (95% CI 40.9–50.5) months in the MSKCC favourable-risk group, 9.0 (95% CI 8.3–9.5) and 24.1 (95% CI 21.9–26.0) months in the MSKCC intermediate-risk group, and 4.5 (95% CI 3.9–6.1) and 9.5 (95% CI 7.2–14.1) months in the MSKCC poor-risk group (PFS: *p* < 0.001 and OS: *p* < 0.001, respectively), respectively. ORR was 33.0% in the favourable-risk group, 24.1% in the intermediate-risk group, and 13.4% in the poor-risk group (*p* < 0.001) (Table 2, Figure 3). Median PFS and OS for patients with one risk factor were 10.1 (95% CI 9.4–11.4) months and 28.2 (95% CI 25.9–30.7) months compared with 6.2 (95% CI 5.5–7.5) months and 16.2 (95% CI 14.5–20.2) months in patients with two risk factors (*p* < 0.001 and *p* < 0.001, respectively). ORR was 26.7% for patients with one risk factor compared with 18.7% for those with two risk factors (*p* = 0.001; Table 2; Figure 4).

## 4. Discussion

The results of the present retrospective, registry-based analysis confirm the relevance of the MSKCC prognostic model in a large cohort of mRCC patients treated with first-line sunitinib in real-life clinical practice. Moreover, within the intermediate-risk group, present data suggest a significantly higher ORR as well as longer PFS and OS for patients with one risk factor as compared with patients with two risk factors.

The MSKCC prognostic model is based on five independent risk factors, including low Karnofsky performance status (KPS), high serum LDH, low haemoglobin level, high corrected serum calcium level, and time from diagnosis to the initiation of systemic therapy of less than one year [3]. The favourable-risk group includes patients with no risk factor, the intermediate-risk group comprises patients with one or two risk factors, and the poor-risk group includes patients with three and more risk factors [3]. The MSKCC model had been proposed and validated in mRCC patients treated with cytokines before the advent of targeted agents [8,9]. In addition to the MSKCC prognostic model, the International Metastatic Renal Cell Carcinoma Database Consortium (IMDC) prognostic model that was subsequently proposed and validated by Heng et al. represents another commonly used tool for the prognostic stratification [10]. In contrast to the MSKCC model, IMDC is based on data of mRCC patients treated with targeted therapies and encompasses six risk factors, that is, low KPS, low haemoglobin level, high platelet count, high neutrophil count, high corrected serum calcium level, and time from diagnosis to the initiation of systemic therapy of less than one year [10]. The MSKCC and IMDC models are both widely used to determine the patient risk group in daily clinical practice as well as in the design and interpretation of clinical trials. The MSKCC model has been prospectively used in the principal clinical trials that introduced targeted agents including sunitinib, pazopanib, bevacizumab, and axitinib [5,11,12,13,14], while the IMDC model has been used in the more recent clinical trials evaluating first-line targeted agents including cabozantinib, or combinations with immune checkpoint inhibitors [15,16,17,18,19]. Both MSKCC and IMDC models are highly concordant with the vast majority of patients being classified into the same risk group. Retrospective studies have validated the feasibility and indicated similar outcomes of patients treated with sunitinib within the stratified prognostic groups [20,21]. The present results showing markedly different outcomes for the favourable-, intermediate-, and poor-risk groups confirmed the feasibility of the MSKCC prognostic model in mRCC patients treated with sunitinib in the real clinical practice, in agreement with prior reports [20,21,22,23]. In the present study, each of the individual MSKCC risk factors had significant association with PFS and OS in the univariable Cox proportional-hazards regression. Among the MSKCC prognostic groups, the intermediate-risk group is numerically the largest, comprising more than one half of all mRCC patients. In the present national cohort, 60.7% of patients were classified as intermediate risk, similarly to previously reported data [20,21,22,23,24,25,26]. The intermediate-risk group is rather heterogeneous because it includes patients with one or two risk factors of different weight, and it had to be determined whether further stratification would improve prognostic performance. Significantly longer PFS (10.7 vs. 6.5 months; *p* < 0.001) and OS (26.3 vs. 14.1 months; *p* < 0.001) for patients with one risk factor compared with patients with two risk factors was reported by Sella et al. in a retrospective analysis of pooled data from six sunitinib clinical trials of 1059 mRCC patients, including 548 MSKCC intermediate-risk patients [27]. Similar results were obtained in the same cohort with the IMDC prognostic model [27]. A difference in prognosis within the MSKCC intermediate-risk group according to the number of positive risk factors has been also suggested by Tamada et al. and Miyazaki et al. in smaller retrospective studies based on the data of 234 and 217 mRCC patients treated with targeted agents, respectively [28,29]. However, these two studies included a limited number of patients, particularly patients in the intermediate-risk group, and patients were treated in different lines and with different targeted agents, introducing heterogeneity as well as a possible bias. The present results are in agreement with the above mentioned reports. A major strength of the present study is the large cohort of patients treated with first-line sunitinib in conditions of the real-life clinical practice. To the best of our knowledge, this is the largest retrospective study reported so far investigating prognostic stratification based on the MSKCC model. Thus, the present results could be used as a benchmark data for the future analyses. Principal limitations include a retrospective design introducing possible selection bias. In particular, current reimbursement criteria in the Czech Republic result in the exclusion of patients with unfavourable prognosis. Another limitation is that only the MSKCC prognostic model was used as the data for the calculation of the IMDC risk score were not available in all patients. Moreover, the MSKCC criteria were slightly modified and total calcium rather than corrected calcium concentration and ECOG PS rather than KPS were used in the present analysis, although these modifications could hardly affect the results.

Biomarkers play a central role in the management of cancer patients [30]. Both MSKCC and IMDC scores are composite prognostic biomarkers combining laboratory and clinical parameters. The systemic treatment during the past than 10 years and more has been dominated by agents targeting the vascular endothelial growth factor (VEGF) pathway. Combinations of anti-VEGF drugs with other targeted agents did not result in increased efficacy [31], and sequential administration of single agents has been established as the principal treatment strategy. With the advent of immunotherapy based on immune checkpoint blockade, the paradigm of sequential therapy has shifted toward combination regimens with immune checkpoint inhibitors [16,17,18]. Although the MSKCC model has been established in the cytokine therapy era and several studies suggested superiority of the IMDC model in patients treated with targeted agents [10,21], the present data indicate, in agreement with other reports, that the MSKCC risk score is still suitable in the targeted therapy era [27,28,29]. However, with the advent of immunotherapy in the first line mRCC treatment, the use of an optimal prognostic model along with the role of prognostic scores will need to be redefined.

## 5. Conclusions

The results of the present retrospective study demonstrate the suitability of the MSKCC model for the prognostic stratification of mRCC patients treated with first-line sunitinib. In addition, within the intermediate-risk group, significantly different outcomes are evident between patients with one or two risk factors. These findings suggest that the number of risk factors should be taken into account during the design of future clinical trials as well as in the data interpretation.

## Figures and Tables

**Table 1:** Patient characteristics based on MSKCC risk groups and details on MSKCC risk factors.

Characteristics	MSKCC Risk Group					
	Favourable	Intermediate	Poor	Intermediate		All Patients
				One Risk Factor	Two Risk Factors	
*n* (%)	806 (33.7)	1450 (60.7)	134 (5.6)	969 (40.5)	481 (20.1)	2390 (100)
Gender, *n* (%)						
Female	225 (27.9)	405 (27.9)	37 (27.6)	261 (26.9)	144 (29.9)	667 (27.9)
Male	581 (72.1)	1045 (72.1)	97 (72.4)	708 (73.1)	337 (70.1)	1723 (72.1)
Age at diagnosis (yr): median	57.9	61.5	62.6	60.8	62.8	60.3
Histology, *n* (%)						
Clear cell carcinoma	772 (95.8)	1365 (94.1)	125 (93.3)	908 (93.7)	457 (95.0)	2262 (94.6)
Papillary carcinoma	30 (3.7)	72 (5.0)	7 (5.3)	53 (5.5)	19 (4.0)	109 (4.5)
Chromophobe carcinoma	2 (0.3)	6 (0.4)	1 (0.8)	5 (0.5)	1 (0.2)	9 (0.4)
Bellini duct carcinoma	1 (0.1)	5 (0.3)	0 (0)	2 (0.2)	3 (0.6)	6 (0.3)
Oncocytoma	1 (0.1)	1 (0.1)	0 (0)	1 (0.1)	0 (0)	2 (0.1)
Unknown	0 (0)	1 (0.1)	1 (0.7)	0 (0)	1 (0.2)	2 (0.1)
Stage at diagnosis, *n* (%)						
I	209 (25.9)	139 (9.6)	1 (0.7)	117 (12.1)	22 (4.6)	349 (14.6)
II	162 (20.1)	144 (9.9)	2 (1.5)	119 (12.3)	25 (5.2)	308 (12.9)
III	180 (22.3)	251 (17.3)	15 (11.2)	176 (18.2)	75 (15.6)	446 (18.7)
IV	110 (13.6)	791 (54.6)	112 (83.6)	464 (47.9)	327 (68.0)	1013 (42.4)
Unknown	145 (18.0)	125 (8.6)	4 (3.0)	93 (9.6)	32 (6.7)	274 (11.5)
Primary tumour grade, *n* (%)						
G1	76 (9.4)	98 (6.8)	8 (6.0)	69 (7.1)	29 (6.0)	182 (7.6)
G2	342 (42.4)	459 (31.7)	45 (33.6)	327 (33.7)	132 (27.4)	846 (35.4)
G3–4	219 (27.2)	646 (44.6)	63 (47.0)	397 (41.0)	249 (51.8)	928 (38.8)
Unknown	169 (21.0)	247 (17.0)	18 (13.4)	176 (18.2)	71 (14.8)	434 (18.2)
**MSKCC Risk Factors**						
ECOG PS 0–1, *n* (%)	806 (100)	1347 (92.9)	94 (70.1)	920 (94.9)	427 (88.8)	2247 (94.0)
ECOG PS ≥ 2, *n* (%)	0 (0)	103 (7.1)	40 (29.9)	49 (5.1)	54 (11.2)	143 (6.0)
Serum calcium ≤ 2.5 mmol/l, *n* (%)	806 (100)	1325 (91.4)	71 (53.0)	907 (93.6)	418 (86.9)	2202 (92.1)
Serum calcium > 2.5 mmol/l, *n* (%)	0 (0)	125 (8.6)	63 (47.0)	62 (6.4)	63 (13.1)	188 (7.9)
Haemoglobin normal, *n* (%)	806 (100)	887 (61.2)	12 (9.0)	747 (77.1)	140 (29.1)	1705 (71.3)
Haemoglobin < LLN, *n* (%)	0 (0)	563 (38.8)	122 (91.0)	222 (22.9)	341 (70.9)	685 (28.7)
Time from diagnosis to therapy initiation ≥ 1 year, *n* (%)	806 (100)	444 (30.6)	11 (8.2)	384 (39.6)	60 (12.5)	1261 (52.8)
Time from diagnosis to therapy initiation < 1 year, *n* (%)	0 (0)	1006 (69.4)	123 (91.8)	585 (60.4)	421 (87.5)	1129 (47.2)
LDH ≤ 1.5 time ULN, *n* (%)	806 (100)	1316 (90.8)	56 (41.8)	918 (94.7)	398 (82.7)	2178 (91.1)
LDH > 1.5 time ULN, *n* (%)	0 (0)	134 (9.2)	78 (58.2)	51 (5.3)	83 (17.3)	212 (8.9)

MSKCC = Memorial Sloan–Kettering Cancer Center (MSKCC) score; OS = overall survival; PFS = progression-free survival; *n* = number of included patients, yr = years; G1 = well differentiated; G2 = moderately differentiated; G3-4 = poorly differentiated/undifferentiated; ECOG PS = Eastern Cooperative Oncology Group performance status; LLN = lower limit of normal; ULN = upper limit of normal; LDH = lactate dehydrogenase.

**Table 2:** Overall response rate (ORR), overall survival (OS), and progression-free survival (PFS) results for sunitinib treatment according to MSKCC risk groups.

Outcomes	MSKCC Risk Group					
	Favourable	Intermediate	Poor	Intermediate		All Patients
				One Risk Factor	Two Risk Factors	
Objective Overall Response						
*n*	806	1450	134	969	481	2390
Objective response rate (%) ^†^	33.0	24.1	13.4	26.7	18.7	26.5
	***p*-value < 0.001**			***p*-value = 0.001**		
Complete response (%)	7.4	4.6	0.7	5.8	2.1	5.3
Partial response (%)	25.6	19.5	12.7	20.9	16.6	21.2
Stable disease (%)	35.1	32.9	18.7	33.7	31.2	32.8
Progressive disease (%)	15.9	24.0	39.6	22.7	26.6	22.1
Not evaluable (%)	16.0	19.0	28.4	16.8	23.5	18.5
**Overall Survival (OS)**						
*n*	806	1450	134	969	481	2390
Median survival (months; 95% CI)	44.7 (40.9–50.5)	24.1 (21.9–26.0)	9.5 (7.2–14.1)	28.2 (25.9–30.7)	16.2 (14.5–20.2)	28.5 (26.3–30.5)
	***p*-value < 0.001**			***p*-value < 0.001**		-
1-year survival (%; 95% CI)	85.0 (82.4–87.6)	69.1 (66.6–71.7)	44.3 (35.0–53.7)	74.3 (71.4–77.2)	58.0 (53.1–62.9)	73.3 (71.4–75.2)
3-year survival (%; 95 %CI)	57.3 (53.4–61.3)	37.1 (34.1–40.1)	9.6 (3.1–16.2)	42.0 (38.3–45.7)	26.3 (21.4–31.2)	42.9 (40.5–45.2)
5-year survival (%; 95% CI)	35.6 (31.2–40.1)	23.4 (20.4–26.4)	3.2 (0.0–8.8)	26.5 (22.7–30.3)	16.5 (11.8–21.2)	26.8 (24.3–29.2)
10-year survival (%; 95% CI)	13.5 (8.1–18.9)	13.8 (10.5–17.0)	0 (0.0–0.0)	15.1 (11.0–19.1)	0 (0.0–0.0)	12.7 (9.8–15.7)
**Progression-Free Survival (PFS)**						
*n*	806	1450	134	969	481	2390
Median survival (months; 95% CI)	17.0 (15.4–18.8)	9.0 (8.3–9.5)	4.5 (3.9–6.1)	10.1 (9.4–11.4)	6.2 (5.5–7.5)	10.6 (9.9–11.5)
	***p*-value < 0.001**			***p*-value < 0.001**		-
1-year survival (%; 95% CI)	61.8 (58.3–65.3)	40.7 (38.0–43.4)	20.8 (13.1–28.5)	45.1 (41.8–48.4)	31.6 (27.1–36.1)	46.9 (44.7–49.0)
3-year survival (%; 95%CI)	25.1 (21.7–28.5)	13.0 (11.0–15.1)	1.8 (0.0–5.1)	15.9 (13.2–18.6)	7.0 (4.2–9.7)	16.6 (14.9–18.4)
5-year survival (%; 95% CI)	10.4 (7.7–13.2)	8.0 (6.2–9.8)	1.8 (0.0–5.1)	9.7 (7.3–12.0)	4.5 (2.1–6.9)	8.4 (7.0–9.9)
10-year survival (%; 95% CI)	3.7 (1.2–6.2)	0 (0.0–0.0)	0 (0.0–0.0)	0 (0.0–0.0)	0 (0.0–0.0)	3.7 (2.1–5.3)

† Complete response + partial response together, CI = confidence interval, MSKCC = Memorial Sloan–Kettering Cancer Center (MSKCC) score, OS = overall survival, PFS = progression-free survival, *n* = number of included patients.
